# Porous Zirconia/Magnesia Ceramics Support Osteogenic Potential In Vitro

**DOI:** 10.3390/ma14041049

**Published:** 2021-02-23

**Authors:** Oleg Prymak, Lida E. Vagiaki, Ales Buyakov, Sergei Kulkov, Matthias Epple, Maria Chatzinikolaidou

**Affiliations:** 1 Inorganic Chemistry, Center for Nanointegration Duisburg-Essen (CeNIDE), University of Duisburg-Essen, 45117 Essen, Germany; matthias.epple@uni-due.de; 2 Department of Materials Science and Technology, University of Crete, 70013 Heraklion, Greece; lidavagiaki@iesl.forth.gr; 3 Foundation for Research and Technology Hellas-Institute of Electronic Structure and Laser (FORTH-IESL), 70013 Heraklio, Greece; 4 Tomsk State University and ISPMS RAS, 634021 Tomsk, Russia; ales.buyakov@gmail.com (A.B.); kulkov@ispms.ru (S.K.)

**Keywords:** functional Mg-based biomaterials, biocompatibility, magnesia and zirconia scaffolds, atomic substitution, Rietveld refinement, microstress, Young modulus, cell adhesion and proliferation, calcium production, collagen secretion

## Abstract

Porous zirconia (ZrO_2_), magnesia (MgO) and zirconia/magnesia (ZrO_2_/MgO) ceramics were synthesised by sintering and designated as ZrO_2_(100), ZrO_2_(75)MgO(25), ZrO_2_(50)MgO(50), ZrO_2_(25)MgO(75), MgO(100) based on their composition. The ceramic samples were characterised by means of scanning electron microscopy, X-ray diffraction, energy-dispersive X-ray spectroscopy and atomic absorption spectrometry to explore the incorporation of Mg atoms into the zirconia lattice. The resulting porosity of the samples was calculated based on the composition and density. The final porosity of the cylinder-shaped ceramic samples ranged between 30 and 37%. The mechanical analysis exhibited that the Young modulus increased and the microstress decreased with increasing magnesia amount, with values ranging from 175 GPa for zirconia to 301 GPa for magnesia. The adhesion, viability, proliferation and osteogenic activity of MC3T3-E1 pre-osteoblastic cells cultured on the zirconia/magnesia ceramics was found to increase, with the magnesia-containing ceramics exhibiting higher values of calcium mineralisation. The results from the mechanical analysis, the ALP activity, the calcium and collagen production demonstrate that the zirconia/magnesia ceramics possess robust osteoinductive capacity, therefore holding great potential for bone tissue engineering.

## 1. Introduction

Zirconia (ZrO_2_) ceramic materials are widely used in clinical applications in the form of load-bearing and wear-resistant implants such as in bone regeneration since they are biocompatible and possess high mechanical strength [1,2]. Depending on the heat treatment, zirconia can be organised in three different crystallographic forms (polymorphism), e.g., monoclinic (ambient temperature up to 1170 °C), tetragonal (1170 to 2370 °C) and cubic (from 2370 to 2700 °C) phases [3]. By mixing ZrO_2_ with other metallic oxides such as magnesia (MgO), calcia (CaO) or yttria (Y_2_O_3_), an increased stability of tetragonal and/or cubic phase can be obtained at lower temperatures [4].

Stabilized zirconia with metal oxides is more favourable compared to pure zirconia because the latter undergoes a tetragonal-monoclinic phase transformation at 1170 °C and this process is accompanied with a volumetric change in the crystal that leads to sudden failure of the material when it cools down following sintering [5]. Stabilised zirconia has a tetragonal phase up to ambient temperature, and this is determinant for its superior mechanical properties. Among the zirconia-based ceramic materials, yttria-stabilised zirconia (YSZ) also known as tetragonal zirconia polycrystal (TZP) is the most studied and widely used combination with excellent resistance to crack propagation [2]. Noticeably, magnesia-stabilised zirconia (MSZ) is also biocompatible and exhibits high mechanical strength and excellent corrosion resistance without experiencing phase transformation, and thus it was found to have better degradation resistance in vivo [6].

Magnesium (Mg)-based alloys have been used successfully in clinical application such in degradable vascular stents. However, the development of Mg-based biomaterials as orthopaedic grafts is hindered due to unpredictable corrosion, and restricted understanding of the tissue response to Mg grafts [7]. Magnesium-based bioceramics include oxides, silicates and phosphates applied in orthopaedic materials such as scaffolds, bone cements or coatings for bone grafts [8]. The number of literature reports on magnesia ceramics for bone regeneration is limited, and they focus mainly on the use of magnesia as an additive or coating compound. For instance, the addition of magnesium oxide at a concentration of 3 wt.% has been reported to significantly enhance the mechanical properties and wear performance of zirconia composites [9], with the magnesium oxide based graft demonstrating the highest Young modulus, hardness, and fracture toughness, and the lowest wear rate. The use of magnesia as additive in calcium phosphates (CaPs) allows for the modification of physical and mechanical properties, the dissolution of CaPs and the improvement of bone formation and biological responses, as reported for the addition of magnesia to *β*-TCP for the fabrication of dense discs followed by in vitro assessment using pre-osteoblasts [10].

Grafted-MgO whiskers are low-cost, easily available materials with high mechanical strength and modulus. A lot of attention has recently attracted their introduction into polymer-based composites leading to reinforcement [11]. Specifically, composites containing grafted-MgO whiskers have been reported to significantly enhance the strength and toughness of a poly(L-lactide) (PLLA), while the alkaline grafted-MgO whiskers could neutralise the acidity of degradation products of PLLA, and regulate the degradation rate of PLLA providing great support in repairing bone defect. Another study using 20 wt.% of magnesia in samples containing calcia, silica, and different additions of zirconia, investigated the in vitro bioactivity of these specimens by analysing their ability to form apatite in simulated body fluid for a period of 7 days. The results indicate that specimens containing 5 and 15 wt.% zirconia were covered by precipitated hydroxyapatite with the characteristic ‘cauliflower’ morphology [12]. Finally, a magnesia-partially stabilised zirconia (MgO-PSZ) ceramic surface treated with CO_2_ laser revealed a more favourable human foetal osteoblastic cell response compared to the non-treated surface, making the laser treated MgO-PSZ surface beneficial for osseointegration at the implant and bone interface [13].

Although magnesium has been used as a degradable biomaterial in the late 19th century [7], the existing literature on the use of magnesia as scaffolds for bone regeneration is very limited. This study presents for the first time a systematic investigation of five scaffold compositions comprising magnesia and zirconia and their in vitro osteogenic responses, focusing on the fabrication, structural and mechanical characterisation of magnesia-stabilized (MS) zirconia, magnesia and MS-zirconia/magnesia ceramics with porosity up to 37% and different weight compositions of MS-ZrO_2_/MgO (100/0, 75/25, 50/50, 25/75 and 0/100 wt.%). A special attention was given to the microscopic, crystallographic and spectroscopic study of produced ceramics to explore the incorporation of Mg atoms into the ZrO_2_ lattice and calculate the resulting porosity of the samples depending on the composition and densities. Moreover, we investigated the pre-osteoblastic cellular responses on the five different ceramic compositions and compared the adhesion, morphology, proliferation and osteogenic differentiation potential of the pre-osteoblastic MC3T3-E1 cells on them.

## 2. Materials and Methods

### 2.1. Fabrication of Porous Zirconia/Magnesia Ceramics

The powders zirconia + NN wt.% MgO (impurities Si, Fe, Cr, Ni, Ti; 0.001–0.01 wt.% and specific surface area of 10.2 m^2^/g) were purchased from the Siberian Enterprise Chemical Group (Certificate No. TU-2320-001-07622928-96, Siberian Enterprise Chemical Group, Seversk, Russia), and processed by plasma-sprayed pyrolysis and chemical co-precipitation in the case of ZrO_2_ (with 3 mol.% MgO), as previously described [14]. The average particle size for both ZrO_2_ and MgO was approximately 500 ± 75 nm, while the particles comprised grains of approximately 20 nm. For the fabrication of the porous zirconia/magnesia composites, zirconia and magnesia powders were mixed at concentrations of 100 wt.% ZrO_2_, 75 wt.% ZrO_2_-25 wt.% MgO, 50 wt.% ZrO_2_-50 wt.% MgO, 25 wt.% ZrO_2_-75 wt.% MgO, 100 wt.% MgO by milling for 25 h to avoid agglomeration and increase homogeneity. Slurries were produced from the milled powders and oleic acid as dispersant. Polyethylene particles with a size ranging from 50–150 µm were added at 20 vol% as pore formers into the mixtures of zirconia, magnesia or zirconia/magnesia at different concentrations in order to create macroporous.

The mixtures were pressed using a hydraulic press at 100 MPa in steel die moulds to form discoid-shaped (d = 15 mm in diameter, h = 3 mm in height) specimens. Polyethylene beads were burned in an oven at 300 °C for 3 h. The organic material was extracted by thermal treatment, generating a desirable porosity in the microstructure. Sintering was carried out in air at 1600 °C in LHT 02/17 High-Temperature Furnaces (Nabertherm GmbH, Lilienthal, Germany) with an isothermal exposure time of 2 h resulting in a final shrinkage of the samples up to d ≈ 12 mm and h ≈ 2 mm.

### 2.2. Characterisation of the Porous Zirconia/Magnesia Scaffolds

A scanning electron microscope (SEM, Apreo S LoVac, Thermo Fisher Scientific, Waltham, MA, USA) with an accelerating voltage of 15 kV was used for the observation of the pore morphology and grain size of ZrO_2_/MgO ceramics and to determine the elemental composition with EDX spectroscopy (Thermo Scientific UltraDry silicon drift X-ray detector, Waltham, MA, USA) operating in a high vacuum with gold/palladium-sputtered samples. For studies on the local distribution of elements (Zr, Mg, O) in the grains, EDX mapping of ceramics was performed. X-ray powder diffraction (XRD) was performed by means of a Bruker D8 Advance instrument (CuKα, 1.54 Å, 40 kV and 40 mA) for qualitative (Diffrac.Suite EVA V1.2, Bruker, Billerica, MA, USA) and quantitative Rietveld (TOPAS 5.0, Bruker) phase analysis. The ceramics were first mortared into fine powder and deposited on a silicon single crystal sample holder. The rotated samples were measured in Bragg–Brentano geometry from 10 to 90° 2*θ* with a step size of 0.01° and a counting time of 0.6 s at each step to obtain a statistical accuracy of 0.2%. The patterns of monoclinic (#37–1484), tetragonal (#50–1089) and cubic ZrO_2_ (#65-0461) and cubic MgO (#45-0946) from the International Centre for Diffraction Data (ICDD) database were used as references. In the Rietveld refinement phase ratios, lattice parameters, crystallite size and microstrain (using Scherrer [15] and the Stokes and Wilson [16] equations, respectively), atomic substitution and crystallographic densities of ZrO_2_/MgO phases were determined. A needed characterisation of the diffractometer was done by measuring a standard powder sample LaB6 from National Institute of Standards and Technology (NIST, Gaithersburg, MD, USA) (Standard Reference Materials (SRM) 660b; a(LaB6) = 4.15689 Å). After determination of the scaffold (ρscaffold) and material (ρmaterial) density the porosity of the samples was calculated under consideration of the phase ratios and atomic substitution (schematically explained in the results) by using the equation, Equation (1):

Ptotal = (1 − ρscaffold/ρmaterial) × 100
(1)

To evaluate the magnesium content in MgO grains (partially also incorporated in cubic MS-ZrO_2_), the ceramics were dissolved in H_2_SO_4_ acid and investigated by atomic absorption spectrometry (AAS) (Thermo Electron, M-Series Atomic Absorption Spectrometer, Waltham, MA, USA).

Mechanical analysis of the ceramic samples was performed by compression tests by means of an Instron-1185 Universal Testing Machine (Instron Corporation, Norwood, MA, USA) with 100 kN at a strain rate of 5 × 10^−4^ s ± 1. Young’s modulus of the least five cylindrical samples with a diameter of 12 mm and a height of 15 mm was calculated from the stress–strain plots derived from load displacement data. Compression strength was calculated as the maximum load by the cross-section of the cylinders. A linear regime of the stress–strain curves from the compression tests was designated as effective Young modulus (E), corresponding to the angle of a tangent slope to a linear segment of the stress–strain curve. The accuracy stress was better than 0.1 MPa for samples with a cross section of approximately 100 mm^2^.

### 2.3. Cell Culture Maintenance and Cell Seeding on Ceramic Samples

Minimum essential medium alpha-MEM, foetal bovine serum (FBS), penicillin/streptomycin, β-glycerophosphate, ascorbic acid, dexamethasone, trypsin/ethylenediaminetetraacetic acid (EDTA), collagen type I, direct red 80, p-nitrophenyl phosphate, alizarin red S and cetylpyridinium chloride (CPC), were purchased from Sigma (St. Louis, MO, USA); PrestoBlue^®^ viability reagent was purchased from Invitrogen Life Technologies (Carlsbad, CA, USA). The MC3T3-E1 cell line from mouse embryonic calvaria was purchased from DSMZ GmbH (ACC 210, Braunschweig, Germany).

Cells were cultured in alpha-MEM cell culture medium supplemented with 10% foetal bovine serum (FBS) and 1% penicillin/streptomycin. This is defined as the complete culture medium. Following, cells were placed in a 5% CO_2_ incubator at 37 °C in wet atmosphere. When cells reach 90% confluence, they were detached using trypsin/EDTA, counted and seeded onto the five different ceramic substrates. An osteogenic medium consisting of 50 μg/mL ascorbic acid and 10 mM *β*-glycerophosphate was used as a supplement to the complete culture medium for the determination of the alkaline phosphatase activity, the produced collagen, and calcium mineralisation.

All five different ceramic substrates were cleaned for 20 min in 100% ethanol in a sonication bath and sterilised in a steam autoclave at 123 °C for 50 min. After sterilisation, the ceramic disks were placed into 24 well plates, seeded with 1 × 10^5^ cells, then, 500 μL of the complete culture medium was added into each well of the plates, and placed in the incubator with medium change every two days. Cells cultured on the tissue culture treated polystyrene (TCPS) surfaces of the plates were used as control.

### 2.4. Cell Viability and Proliferation Assay

Pre-osteoblastic MC3T3-E1 cells were seeded on the five ceramic samples at a density of 1 × 10^5^ cells/sample in 24-well plates and cultured for 2 and 14 days in a humidified incubator at 37 °C. The viability and proliferation of pre-osteoblastic cells cultured on the ceramic samples was assessed using the PrestoBlue^®^ (Thermo Fisher Scientific, Waltham, MA, USA) cell viability assay according to the manufacturer instructions at a 1:10 dilution in culture medium. The assay is based on the use of a non-toxic solution, resazurin, which, in a reducing environment of living cells is reduced to resorufin. After the addition of the reagent, the plates were placed in the incubator for 30 min. Colour development was detected in a spectrophotometer (Synergy HTX Multi-Mode Microplate Reader, BioTek, Bad Friedrichshall, Germany) at 570/600 nm. OD values were expressed as mean of triplicates of three independent experiments (*n* = 9).

### 2.5. Cell Adhesion and Morphology

Cell adhesion and morphology on the ceramic samples was evaluated by means of scanning electron microscopy (SEM, JEOL JSM-6390 LV, Tokyo, Japan). Pre-osteoblastic cells were seeded on each of the five ceramic samples at a density of 1 × 10^5^ cells/sample in 24-well plates and cultured for 2 and 14 days in a humidified incubator at 37 °C. All samples were fixed by addition of 4% paraformaldehyde (PFA), dehydrated with increasing concentration of 20, 50, 70, 90 and 100% ethanol that was exchanged with liquid carbon dioxide by critical point drying (Baltec CPD 030, Baltec, Los Angeles, CA, USA). The dry samples were sputter-coated with gold (Baltec SCD 050) to increase their conductivity, and were then placed under the scanning electron microscope (JEOL JSM-6390 LV, Tokyo, Japan) for observation at a voltage of 20 kV.

### 2.6. Alkaline Phosphatase (ALP) Activity Measurement

For the alkaline phosphatase (ALP) activity measurement, 1 × 10^5^ pre-osteoblastic cells were seeded on the five ceramic materials and cultured for 7 and 15 days. Cell layers were detached from the substrates with trypsin-EDTA, followed by centrifugation at 1200 rpm for 15 min at 4 °C. Then, the cell pellets were washed twice with phosphate buffer saline (PBS) for 10 min and were resuspended in 100 μL lysis buffer containing 0.1% Triton X-100 in 50 mM Tris-HCl and 50 mM phenylmethylsulfonyl fluoride, pH 10. The pellets were disrupted by two freezing/thawing cycles at −20 °C/room temperature. At the end of the second cycle, an aliquot of 100 μL cells was mixed with 100 μL of 2 mg/mL p-nitrophenyl-phosphate (pNPP) diluted in 50 mM Tris-HCl, pH 10, with 2 mM MgCl_2_, and incubated for 1 h at 37 °C until the reaction was stopped by the addition of 50 μL 1 N NaOH and colour change was measured by means of a spectrophotometer (Synergy HTX Multi-Mode Microplate Reader, BioTek, Bad Friedrichshall, Germany) at 405 nm. The enzymatic activity was calculated using the equation [units = nmol p-nitrophenol/min] and normalised to cellular protein in lysates determined using the Bradford protein concentration assay (AppliChem GmbH, Darmstadt, Germany). Cells seeded on TCPS surfaces were used as control. All substrates were analysed in triplicates, in three independent experiments (*n* = 9).

### 2.7. Extracellular Collagen Assessment

The quantification of secreted collagen levels collected from the supernatants of 1 × 10^5^ pre-osteoblastic cell cultures on different ceramic samples was performed by means of a colorimetric assay based on the selective binding of Sirius Red dye in acid solution to the proline-hydroxyproline-glycine sequence of the helical structure of collagen [17]. For the determination and quantification of collagen, 1 mL of dye solution at a concentration of 0.001 g/mL was added to each sample, followed by centrifugation for 15 min at 15,000 rpm at 4 °C. The dye was decanted from the tubes carefully to avoid dissolving the pellet, 500 μL of 0.1 N HCl were added to each sample, and centrifuged for 15 min at 15,000 rpm. Next, the HCl was removed and replaced by 500 μL 1 N NaOH. The samples were stirred thoroughly until the pellet was dissolved. Finally, the aliquots of each sample were placed in a 96-well plate and the absorption was measured at 530 nm in a spectrophotometer (Synergy HTX Multi-Mode Microplate Reader, BioTek, Bad Friedrichshall, Germany). Culture medium was used as a blank. The amount of collagen content was calculated based on a linear standard curve constructed from known concentrations of collagen type I. Cells seeded on TCPS surfaces were used as control. All substrates were analysed in triplicates, in three independent experiments (*n* = 9).

### 2.8. Alizarin Red Staining of Calcium Deposits

Calcium deposits, a late marker of osteogenesis signalling the formation of extracellular matrix, were stained with alizarin red. Before staining, the samples were evaluated by means of the PrestoBlue^®^ viability assay for normalisation. After the cell viability assessment, the samples were rinsed with PBS and fixed with 4% paraformaldehyde for 15 min. After removing paraformaldehyde, cells were rinsed twice with PBS and stained with 500 μL of 2% solution of alizarin red dye in water. To remove the excess dye, samples were thoroughly washed with deionized water. For quantification, each stained sample was extracted by incubation in 500 μL 10% cetylpyridinium chloride in 10 mM Na_2_PO_4_, pH 7.0, for 1 h at room temperature under shaking. A volume of 200 μL was transferred into a 96-well plate prior to reading and the absorbance was measured at 550 nm. The OD values from the extraction were normalized to 10^6^ cells from the viability assay. Cells seeded on TCPS surfaces were used as control. All substrates were analysed in triplicates, in three independent experiments (*n* = 9).

### 2.9. Statistical Analysis

Cell viability, collagen production, alkaline phosphatase activity and calcium biomineralisation data are presented as mean values ± standard deviation. Statistical analysis was performed by means of GraphPad Prism version 8 software using one-way analysis of variance and Tukey’s multiple comparison to evaluate the significant differences among means of values obtained from all five ceramic compositions as well as of TCPS control at each experimental time point. A p < 0.05 was considered significant, if no other indication is stated.

## 3. Results

### 3.1. Physicochemical, Morphological and Mechanical Properties of the Porous Zirconia and Magnesia Ceramics

**Figure 1:**
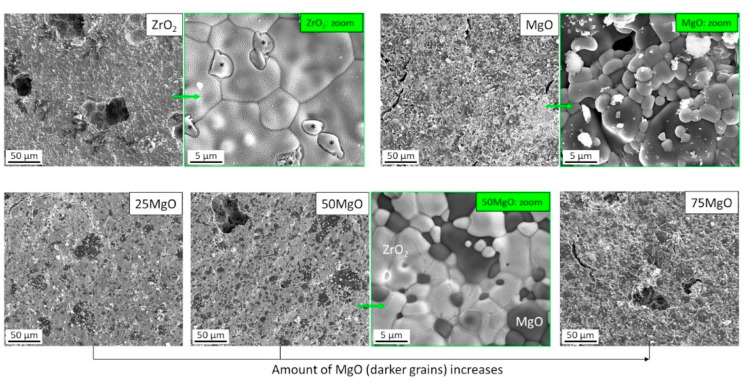
SEM micrographs of pure (**top**) and mixed (**bottom**) ZrO_2_/MgO ceramics. Lighter (ZrO_2_) and darker (MgO) grains are well visible. Additional formations (*) in pure ZrO_2_ are probably the tetragonal t-ZrO_2_ phase (see text).

**Figure 2:**
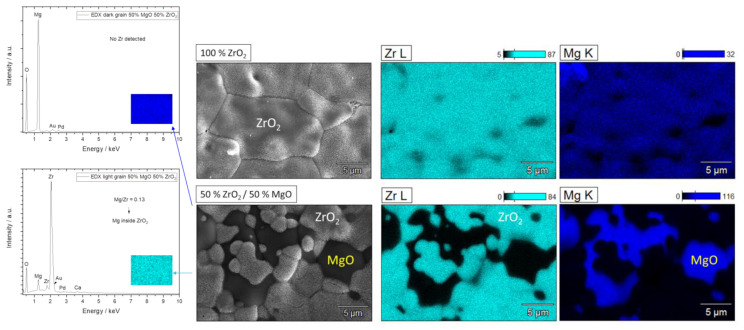
Representative EDX mapping of pure 100% ZrO_2_ (**top**) with a homogeneous distribution of Zr/Mg inside the grains and mixed 50% ZrO_2_/50% MgO (**bottom**) ceramics with well detectable ZrO_2_ (Mg is incorporated) and MgO grains.

The microstructure of produced ceramics was investigated by SEM. SEM analysis displayed that the samples consisted of microcrystalline well interconnected grains with a size of about 5 µm and contained pores with an average size of 50–60 µm (Figure S1). Due to the different contrasts of ZrO_2_ (lighter) and MgO (darker) grains in SEM (Figure 1), caused by the atomic numbers of Zr (Z = 40) and Mg (Z = 12), it was possible to distinguish between both the oxides as also confirmed by EDX (Figure 2). Furthermore, it was observed that the amount of darker MgO grains increased with the increasing MgO content in the ZrO_2_/MgO ceramics in a good agreement with the nominal composition. The surface of the grains also differed from each other, e.g., whereas MgO remained smooth, magnesia-stabilized MS-ZrO_2_ showed a brain-like relief as for the pure ZrO_2_ and for the mixed ZrO_2_/MgO ceramics. Additionally to the found main grains, a small amount of elongated (Figure S1) or flat (Figure 1) crystals were also observed.

To investigate the elemental distribution in the grains, EDX mapping on pure 100% ZrO_2_ and mixed 50% ZrO_2_/50% MgO ceramics was performed (Figure 2). The results exhibited a homogeneous Mg/Zr distribution inside the MS-ZrO_2_ grains. As observed the darker grains belonged to MgO without any incorporated Zr atoms, whereas ZrO_2_ grains contained Mg indicating a Zr/Mg substitution in ZrO_2_ (molar ratio Mg/Zr = 0.13). In agreement to SEM and nominal compositions of the samples, EDX data further confirmed the increasing tendency of MgO with the exponentially varying molar Mg/Zr ratios (Figure S2). A small amount of Mg was found in 100% ZrO_2_ as expected to be in magnesia-stabilized zirconia, whereas no Zr was detected in MgO. A small peak of Ca can be attributed to impurities during annealing of slurries, oleic acid as dispersant and polyethylene particles. It should be noted that the composition of 25% MgO sample was corrected to 35% as determined by XRD (Table 1).

**Figure 3:**
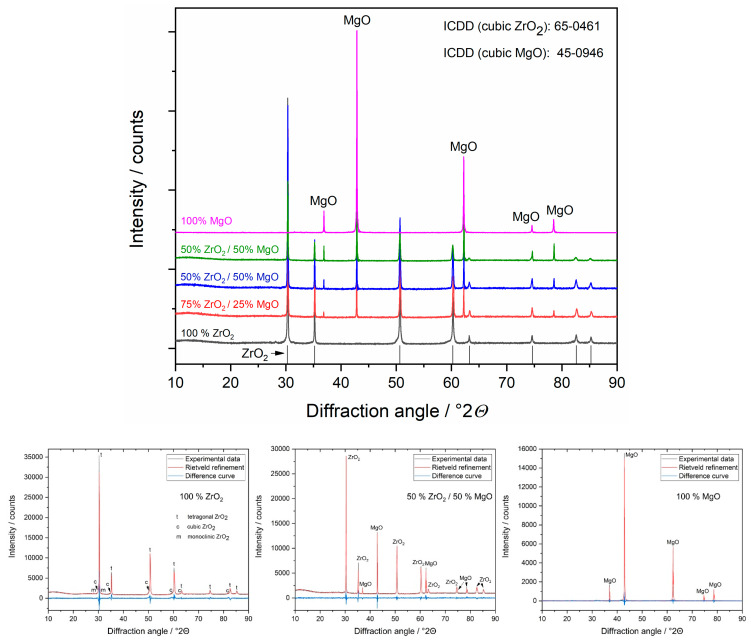
Representative X-ray diffractograms (**top**) and Rietveld refinements (**bottom**) of ZrO_2_/MgO ceramics. The phases were identified using the references from the ICDD database. In the ZrO_2_ ceramic different ZrO_2_ phases are detectable: tetragonal (t), cubic (c) and monoclinic (m), whereas in the mixed samples only c-ZrO_2_.

XRD measurements and following Rietveld refinements provided information about the crystalline structure of the samples (Figure 3). It was shown that 100% ZrO_2_ ceramic contains two main phases, tetragonal (t, 66 wt.%) and cubic (c, 31 wt.%), and minor monoclinic (m, 3 wt.%) phases (see Table 1). This agrees well with the ZrO_2_-MgO phase diagram [18] and applied heat treatment (1600 °C) of magnesia-stabilised ceramic (MS, ≈3 mol% MgO) leading to the formation of major two-phase system of t-ZrO_2_/c-ZrO_2_ and small amount of m-ZrO_2_ due to cooling to room temperature. The determined lattice parameters indicate an incorporation of Mg (≈4 at.%) into the c-ZrO_2_ lattice, whereas Mg atoms in the t- and m-ZrO_2_ phases were not detectable by XRD. This matched again the larger Mg-miscibility in c-ZrO_2_ and low miscibility in t- and m-ZrO_2_ phases and the finding by EDX. The temperature-induced phase transformation to m-ZrO_2_ was accompanied by a volume increase of 6% (from 132.2 to 140.5 Å3) inducing compressive (residual) stress in c-ZrO_2_ and atomic defect concentration quantified in XRD by microstrain (ε = 0.34 %), which was significantly larger than that of t- and m-ZrO_2_ (ε = 0.08 and 0.05%, respectively). Such c-t-m transformation further contributes to the mechanical properties of ZrO_2_-based ceramics, known as transformation toughening [19], which would influence their application in medicine [20]. The XRD results also showed that both c-/m-ZrO_2_ were nanocrystalline (CS ≈ 40–50 nm), whereas t-ZrO_2_ was microcrystalline (Table 1), which is in agreement with SEM observations (Figure 1).

**Table 1:** Crystallographic parameters determined by Rietveld refinement in XRD for ZrO_2_/MgO ceramics. The porosity of the samples was calculated considering both the density (incl. effect of Zr/Mg substitution) and the weight amount of each phase (see explanation in Figure 4).

Nominal Composition	0% MgO/100% ZrO_2_			25% MgO/75% ZrO_2_		50% MgO/50% ZrO_2_		75% MgO/25% ZrO_2_		100% MgO/0% ZrO_2_
Phases	Cubic (ZrMg)O_2_	Tetragonal ZrO_2_	Monoclinic ZrO_2_	Cubic (ZrMg)O_2_	Cubic MgO	Cubic (ZrMg)O_2_	Cubic MgO	Cubic (ZrMg)O_2_	Cubic MgO	Cubic MgO
**Phase ratio/wt.%**	31	66	3	65	35	50	50	26	74	100
**Lattice** **parameters/Å**	a = 5.0944 (4)	a = 3.5928 (1) c = 5.0888 (3)	a = 5.13 (1) b = 5.21 (1) c = 5.32 (1) β = 99.1 °	a = 5.0804 (1)	a = 4.2126 (1)	a = 5.0865 (1)	a = 4.2123 (1)	a = 5.0911 (1)	a = 4.2126 (1)	a = 4.2137 (1)
**Unit cell V/Å^3^**	132.21 (3)	65.69 (6)	140.5 (1)	131.12 (1)	74.76 (1)	131.60 (1)	74.74 (1)	131.96 (1)	74.76 (1)	74.84 (1)
**Crystallite size *CS*/nm**	45 (3)	µm	39 (9)	µm	µm	µm	µm	µm	µm	162 (2)
**Microstrain *ε*/%**	0.34 (1)	0.08 (1)	0.05 (1)	0.08 (1)	0.02 (1)	0.08 (1)	0.02 (1)	0.10 (1)	0.02 (1)	0.00
**Density *ρ*/g/cm^3^**	5.90	6.14	5.83	5.57	3.58	5.75	3.58	5.84	3.58	3.58
**Mg in cubic ZrO_2_/at.%**	≈4	-	-	≈15	-	≈9	-	≈6	-	-
**Mg/Zr molar ratio**	0.04	-	-	0.18	-	0.10	-	0.06	-	-
**Porosity/%**	≈33			≈30		≈31		≈33		≈37

**Figure 4:**
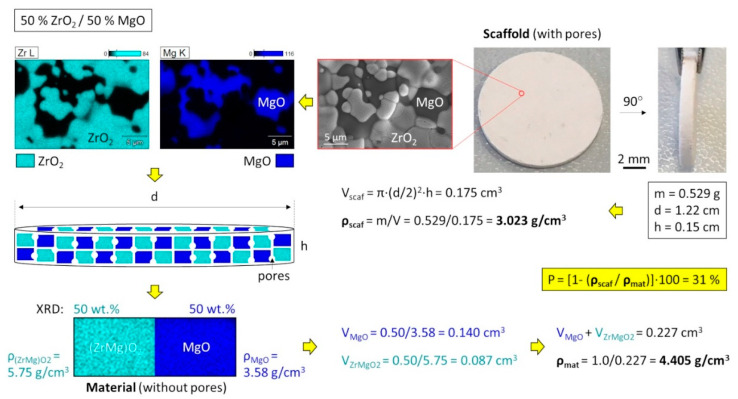
Calculation of the porosity for a two-phase system considering the crystallographic densities (caused by different atomic substitutions) and phase ratios exemplary shown for 50% ZrO_2_/50% MgO.

Crystallographic study confirmed that the mixed ceramic samples with 25, 50 and 75% MgO consisted of c-ZrO_2_ and MgO phase (Table 1) as also expected from the phase diagram for these compositions. It should be noted that a small amount of the low-temperature m-ZrO_2_ phase (~1%) is possible as also assumed from the elongated crystals in SEM (Figure S1), but its detection by XRD is behind of resolution. The determined phase ratios agreed well with the nominal one, with an only small difference for 25% MgO (calculated by XRD 35 wt.%). All phases were microcrystalline which correspond to the grain size of MgO and ZrO_2_ observed by SEM (Figure 1). The lattice parameters of c-ZrO_2_ increased with increasing MgO content indicating an opposite trend in the Zr/Mg substitution (15, 9, 6 at.%, respectively). The larger Zr/Mg substitution ratio for the mixed ceramics compared to 100% ZrO_2_ (4 at.%) can be explained by the compositional location (right or left from the miscibility area of c-ZrO_2_) in the phase diagram. The determined microstrain of c-ZrO_2_ remained almost constant (ε = 0.08%) for all samples, which pointed on the pre-stressed condition of this phase compared to MgO (ε = 0.02%) or m-ZrO_2_ (ε = 0.05%).

A special attention was given to the calculation of porosity of the ceramics because they contained several phases with different material densities caused by the incorporated Mg atoms in the ZrO_2_ structure (Table 1). Considering all these factors, it was important to optimize the equation of porosity and correctly applied them in the calculation, as exemplary shown for 50% ZrO_2_/50% MgO cylindrical sample with a dimension d = 12.2 mm and h = 1.5 mm (Figure 4). Here it is to mention that material densities were not additive and the mean value can be determined by a division of the total mass (here normalized to 1) by the total volume of the phases. Thus, the ZrO_2_ containing ceramics showed a porosity of ~30–33%, whereas pure MgO ceramic was more porous (37%), which agreed well with the microscopic investigation by SEM (Figure S1).

To verify a slight deviation in the composition (nominal 25 wt.% MgO) from the linear rule determined by XRD (Figure S3, left), AAS measurements in H_2_SO_4_ acid dissolved ceramics were performed and the same discrepancy was found (Figure S3, right). The results also provided underestimated values for the Mg^2+^ content (25% smaller than theoretically possible) indicating an incomplete solubility of MgO [21] and partial protection of remaining Mg in the (Zr, Mg)O_2_ phase due its slow solubility [15].

**Figure 5:**
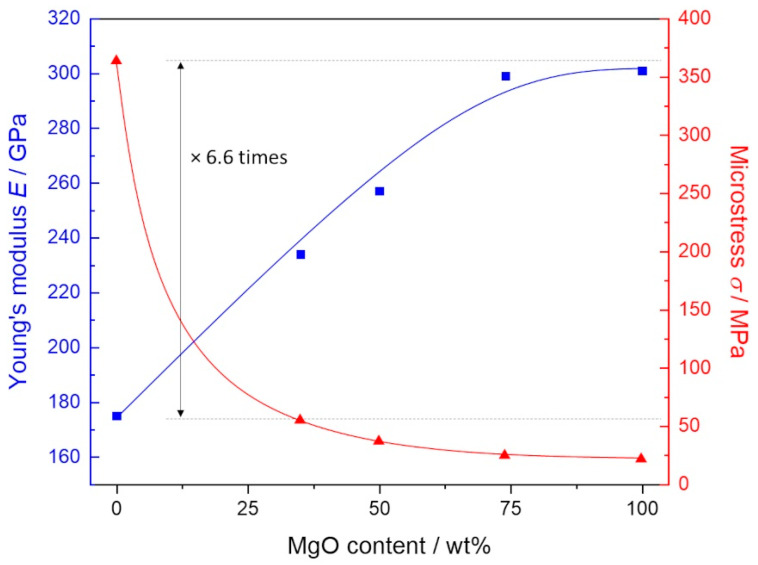
Mechanical properties of ZrO_2_/MgO ceramics showing an increasing Young‘s modulus (*E*) and decreasing microstress (σ) followed by increasing MgO content. Microstress of 100 % ZrO_2_ is ~6.6 times larger than that of ZrO_2_/MgO ceramic.

The mechanical analysis presented that the transformation toughening in magnesia stabilised (MS) zirconia can be also affected by varying MgO content in the ZrO_2_/MgO ceramics (Figure 5). It is observed that the determined Young modulus (*E*) increased while the microstress (*σ*) decreased with an increasing MgO amount. Interestingly, the addition of more than 25 wt.% MgO contributed to a significant drop in microstress compared to MS-ZrO_2_ following a functional dependency similar to the Zr/Mg molar ratio determined by EDX (Figure S2). This fact may be due to a possibility of MgO for small plasticity and relaxation therefore microstresses. Furthermore, the found drop (6.6 times) corresponded well to a decrease in microstrains for c-ZrO_2_ (4.3 as from 0.34/0.08) for these compositions (Table 1). Simultaneously, the Young modulus continuously increased from 175 GPa (ZrO_2_) to 301 GPa (MgO) reaching saturation at higher MgO concentration.

### 3.2. Cell Adhesion and Morphology on the Ceramic Samples

**Figure 6:**
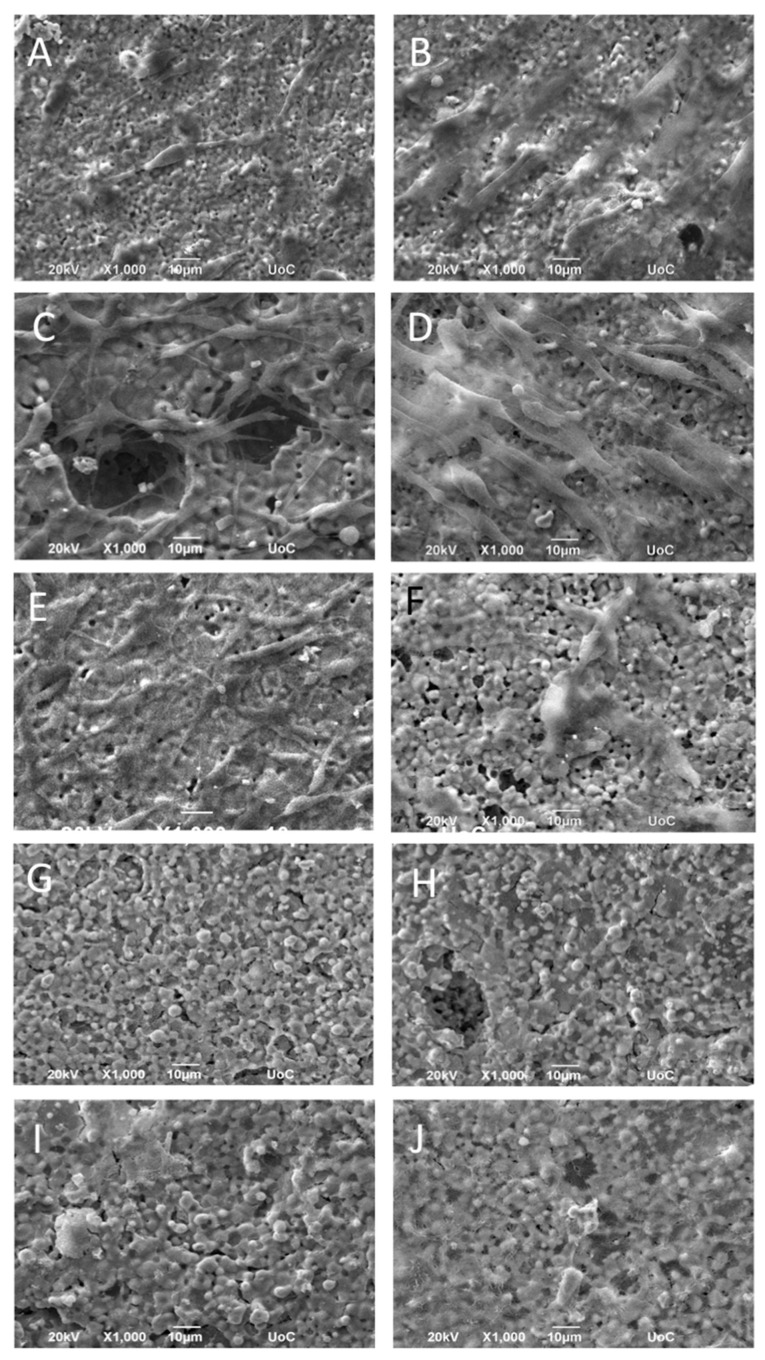
SEM images illustrating MC3T3-E1 cell adhesion and morphology on ZrO_2_(100) (**A**,**B**), ZrO_2_(75)MgO(25) (**C**,**D**), ZrO_2_(50)MgO(50) (**E**,**F**), ZrO_2_(25)MgO(75) (**G**,**H**), and MgO(100) (**I**,**J**) ceramics after 2 (left panel) and 14 days (right panel) in culture at a magnification of 1000-fold. Scale bars represent 10 μm.

The morphology of the MC3T3-E1 pre-osteoblastic cells on the different compositions of zirconia/magnesia ceramics after 2 and 14 days in culture was investigated by scanning electron microscopy, and representative images are depicted in Figure 6. Specifically, Figure 6A,B show an elongated pre-osteoblastic cellular morphology on the ZrO_2_(100) ceramics on days 2 and 14, respectively, with the latter (Figure 6B) indicating an increased number of cells proliferated after the period of two weeks. A similar characteristic elongated cell morphology with visible cell protrusions was obtained on ZrO_2_(75)MgO(25) ceramic samples (Figure 6C,D) on both time points, as well as on ZrO_2_(50)MgO(50) samples (Figure 6E,F), on which cells indicated a more flattened morphology after 14 days. On the ceramics with a higher content on magnesia, ZrO_2_(75)MgO(25) and MgO(100) that exhibited a granular structure as illustrated in Figure 6G–J, respectively, we observed adhered cells on days 2 and 14. In these images some cell nuclei are visible. Notably, on the MgO(100) substrates, an intercellular network formation is depicted at both time points, and this is more pronounced after 14 days in culture.

### 3.3. Cell Viability and Proliferation on the Ceramic Samples

**Figure 7:**
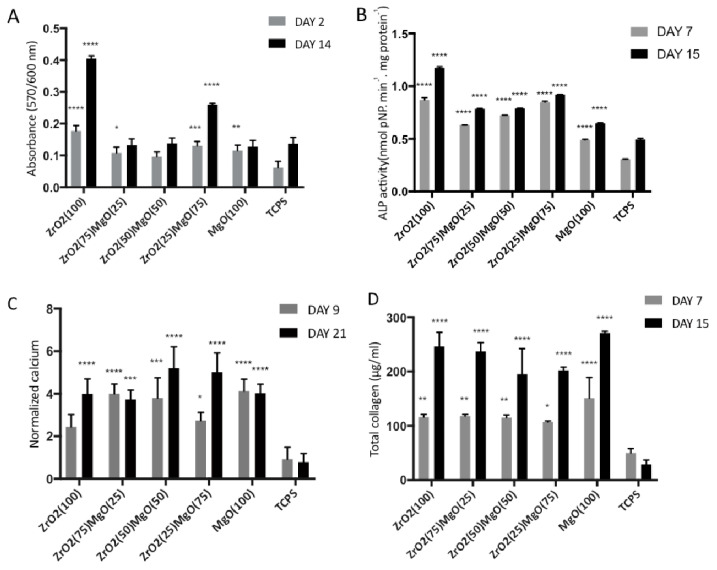
(**A**) Cell viability and proliferation on zirconia and magnesia ceramics after 2 and 14 days of culture, (**B**) alkaline phosphatase activity on the different ceramic substrates on days 7 and 15 in culture, (**C**) normalised calcium biomineralisation of MC3T3-E1 cells cultured for 7 and 14 days on the five ceramic materials after staining with alizarin red and (**D**) levels of total collagen produced by pre-osteoblastic cells cultured on the five ceramic substrates for 7 and 15 days. Data are expressed as mean of triplicate samples ± standard deviation (SD). Asterisks indicate statistical significance compared to the TCPS control surface at each experimental time point (*: *p* < 0.05, **: *p* < 0.01, ***: *p* < 0.001, ****: *p* < 0.0001).

The pre-osteoblastic cell viability on the five compositions of the ceramic materials after 2 and 14 days in culture is presented in Figure 7A. Among the five ceramic compositions, the highest cell viability was found in ZrO_2_(100) at both experimental time periods of 2 and 14 days, while the number of viable cells was similar on the other ceramic substrates, ZrO_2_(75)MgO(25), ZrO_2_(50)MgO(50), ZrO_2_(25)MgO(75) and MgO(100). Statistical analysis indicated that on day 2 the ZrO_2_(100) ceramics presented a significant difference in cell viability compared to all other materials and TCPS control (*p* < 0.02 up to 0.0001). Additionally, ZrO_2_(75)MgO(25), ZrO_2_(25)MgO(75) and MgO(100) reached significantly higher cell viability compared to the TCPS control (*p* < 0.02 up to 0.0001). On day 14, ZrO_2_(100) ceramics displayed a significant difference in cell viability compared to all other materials and TCPS control (*p* < 0.0001), while ZrO_2_(25)MgO(75) revealed significantly higher cell viability compared to all other ceramic materials and the TCPS control (*p* < 0.0001).

### 3.4. Alkaline Phosphatase Activity

Alkaline phosphatase (ALP) activity of cells cultured on the five ceramic substrates was measured after 7 and 15 days, normalised to the total protein concentration, and the results are presented in Figure 7B. The enzymatic activity on day 7 indicated similarly high responses for the zirconia-containing ceramics, with ZrO_2_(100) and ZrO_2_(25)MgO(75) showing the highest response, presenting at least two times higher values compared to TCPS. The ALP activity on MgO(100) was found to be the lowest among the ceramics, but higher than on the TCPS control. A similar pattern was measured on day 15 with the ZrO_2_(100) indicating the highest value, which is 30% higher than on day 7. For all ceramic materials, an increase in the ALP activity was found on day 15, compared to day 7. Statistical analysis revealed that on day 7, all ceramics exhibited significantly different ALP activity values when compared to each other and also to the TCPS control, except of the ZrO_2_(100) vs. ZrO_2_(25)MgO(75) (*p* < 0.0001). Similarly, on day 15, all ceramics displayed significant differences among them (*p* < 0.0001), except of the ZrO_2_(50)MgO(50) vs. ZrO_2_(75)MgO(25).

### 3.5. Matrix Mineralisation by Calcium Production

The levels of calcium produced by the pre-osteoblastic cells cultured on the zirconia/magnesia ceramics on days 9 and 21 are depicted in Figure 7C. All ceramic materials indicated significant difference in the produced calcium when compared to the TCPS control at both time periods of 9 and 21 days, whereas the differences were not found to be significant among the five ceramic materials. On day 9, the highest calcium mineralisation was measured on ZrO_2_(75)MgO(25), ZrO_2_(50)MgO(50) and MgO(100), whereas on day 21 the highest values were found on ZrO_2_(25)MgO(75) and ZrO_2_(50)MgO(50), however not significant.

### 3.6. Collagen Production

To study the effect of porous zirconia/magnesia ceramics on the production of collagen, we quantified the secreted collagen in culture supernatants on days 7 and 15, as shown in Figure 7D. Cells on all five ceramic substrates, with at least 200 μg/mL, produced approximately the double amount of collagen after 15 days in culture, compared to the values after 7 days. The highest secreted collagen values were found to be on the MgO(100) ceramics, at both time periods, however not statistically significant. The differences of the produced collagen values were found to be statistically significant when compared to the TCPS control at both time points of 7 and 15 days, with the latter demonstrating a higher significance (*p* values < 0.0001). Among the five ceramic materials, the differences were not found to be significant.

## 4. Discussion

The focus of this study was the preparation, physicochemical and mechanical characterisation of porous ceramic scaffolds with varying compositions of zirconia and magnesia and the investigation of their osteogenic response. This is one of the few studies employing porous magnesia ceramic scaffolds for bone tissue regeneration, as magnesia is mainly used as an additive or surface coating material and thus there is limited number of reports on magnesia scaffolds in the literature.

Disc-shaped ceramic samples were produced by combining 100, 75, 50, 25 and 0 wt.% magnesia stabilised zirconia (ZrO_2_) with 0, 25, 50, 75, 100 wt.% magnesia (MgO), respectively, and designated as ZrO_2_(100), ZrO_2_(75)MgO(25), ZrO_2_(50)MgO(50), ZrO_2_(25)MgO(75), MgO(100), respectively. The final porosity of the cylinder-shaped samples in this study ranges between 30 and 37%. These variations are due to the lack of control in the level of shrinkage during fabrication.

We observed by means of SEM that the amount of darker MgO grains increased, increasing the MgO content in the zirconia/magnesia ceramics, and this is in a good agreement with the nominal composition. Similar differences in the Z-contrast in SEM have been also observed for ZrO_2_/MgO [16] and ZrO_2_/Al_2_O_3_ [17] ceramics. The surface of the grains are different among ceramics, with magnesia-stabilized MS-ZrO_2_ showing a brain-like structure as for the pure ZrO_2_ and for the mixed zirconia-magnesia ceramics. Such irregular structures with a dendritic size of approximately 100 nm can be attributed to the eutectic growth of the oxides [18,19] due to the specification of the ZrO_2_-MgO phase diagram [20,22]. Apart from the main grains, a small amount of elongated or flat crystals were also observed, which can be related to the remaining parts of other tetragonal and monoclinic ZrO_2_ phases [23,24]. The data from EDX mapping exhibit a homogeneous Mg/Zr distribution within the magnesia-stabilized zirconia grains as it has been previously demonstrated for yttria-stabilized zirconia [25]. In addition, to verify any deviation in the magnesium content from the linear rule determined by XRD, we performed the atomic absorption spectrometry analysis.

Our results show that the Young modulus increased and the microstress decreased with the increasing MgO amount. Specifically, the Young modulus continuously increased from 175 GPa for zirconia to 301 GPa for magnesia in agreement with the literature [26,27] reaching a saturation at higher MgO concentration [8]. A broad variation in microstress from 364 MPa (ZrO_2_) to 21 MPa (MgO) can be of interest in dentistry [28] with different requirements for the mechanical properties of dentin and enamel [29,30,31].

Events occurring on the tissue-material interface control the integration of grafts into bone. It is acknowledged that a strong initial adhesion of osteoblastic cells on biomaterials leads to better bonding between implant and bone [32,33]. By means of SEM we found a good initial adhesion of pre-osteoblastic that displayed an elongated morphology covering the surface of the ZrO_2_(100) ceramics at both days 2 and 14, and a similar cell morphology with visible cell protrusions on ZrO_2_(75)MgO(25) ceramics, as well as on ZrO_2_(50)MgO(50) samples. In a previous report with zirconia ceramics stabilised either with magnesia (MgSZ) or yttria (YSZ), after 1 day in culture, pre-osteoblastic cells adhered well on both zirconia ceramics with a more spread out morphology on YSZ compared to MgSZ [34]. On the ceramics with a higher content of magnesia, ZrO_2_(75)MgO(25) and MgO(100) that depicted a granular structure, we observed a high number of adhered cells covering the samples at day 2. Particularly on day 14, we observed densely adhered interconnected cells on the MgO(100) ceramics. These results are in agreement with the morphology of human pre-osteoblastic cells presented in a previous report on Mg-tricalcium phosphate microstructured ceramics [10] indicating a single layer of cells covering the sample surface at day 3, with similar multiple layers detected at day 9.

Among the five ceramic materials, the highest pre-osteoblastic cell viability was found on ZrO_2_(100) at both day 2 and 14 in culture, indicating the highest proliferation increase after 14 days. Previous reports have revealed the potential of zirconia to promote cell proliferation over three weeks in culture [34]. All other zirconia/magnesia ceramics showed similar proliferative capacity of the pre-osteoblastic cells. It appears that the chemical composition of the ceramics did not affect the cell viability and proliferation, demonstrating the capacity of magnesia ceramics to support cell growth either in combination with zirconia or as pure magnesia. Previous studies have reported on the positive effect of scaffold porosity on cell proliferation, including zirconia and alumina ceramics [25]. In this study, the porosity of the ceramics ranging from 30–37% was not found to affect the cell viability, proliferation and osteogenic potential. Moreover, the porosity allowed for the effective circulation of fluid and transportation of nutrients through the porous structure favouring the migration and proliferation of cells [25].

All five ceramic compositions tested were found to support the osteogenic differentiation markers including the ALP activity, calcium mineralisation and collagen production, demonstrating higher responses compared to the TCPS control. Specifically, we found the highest values of calcium mineralisation on day 21 on ZrO_2_(25)MgO(75) and ZrO_2_(50)MgO(50), and higher calcium in all magnesia-containing ceramics on day 9, the highest secreted collagen on the MgO(100) ceramics at days 7 and 15, and the highest ALP activity on day 7 on ZrO_2_(100) and ZrO_2_(25)MgO(75), however these results were not statistically significant. Our findings demonstrate similar levels of collagen production and higher calcium production on magnesia-containing ceramics compared to another study reporting on pre-osteoblasts cultured on magnesia stabilised zirconia ceramics [34], and show higher calcium mineralisation compared to porous alumina and alumina/zirconia ceramics [14]. Another research group has grafted magnesia and chitosan whiskers into a PLLA matrix and reported on a significant increase in the ALP secretion and calcification of pre-osteoblastic cells compared to the PLLA, indicating that both magnesia and chitosan were advantageous to cell adhesion, proliferation and decrease of cell apoptotic rate [11]. The release of magnesia and chitosan from the composite and its exposure to cell culture medium might induce a microenvironment change leading to alkalisation, which may have a positive effect on cell metabolism. In another study, C2C12 cells were cultured on Mg modified calcium phosphate cement substrates with BMP-2 and demonstrated increased in vitro osteogenic differentiation and phosphorylation of Smad 1/5/8 [35]. In this case, it has been reported that the soluble magnesia ions made little difference to the osteogenic capacity, and that the magnesium on the substrates mediated the adsorption and conformation of rhBMP-2 bound on the matrix, thus promoting the recognition to type I and type II BMP receptors, and increasing osteogenic activity.

The results from the ALP activity, calcium and collagen production clearly demonstrated that the zirconia/magnesia ceramics possess robust osteoinductive capacity, and thus present great potential for bone tissue engineering applications. In addition, ceramic materials can be used as carriers to deliver cytokines such as bone morphogenetic proteins [36], specific osteogenic growth factors that enhance bone formation in vitro [37] and in vivo [33], as well as plasmids for the transfection of bone-forming cells [38], or cement formulations [39] leading to enhanced osteogenic responses. Magnesium-based orthopaedic implants such as pins, plates and screws have been extensively investigated in both small and large animal models, in physiological and osteoporotic models, mainly to observe the degradation pattern of Mg-based implants and the response of peri-implant bone tissue [40]. In addition, animal studies have been performed to evaluate the biological activity of Mg-based alloys in vivo, and based on their tissue regeneration potential, new products such as wound-closing sutures wires have been developed [41]. Our findings on the magnesia-containing ceramic scaffolds demonstrating higher values of calcium mineralisation after three weeks in cell culture maintaining their initial shape, have great potential to elicit a good osteogenic response in vivo.

## 5. Conclusions

In this study, we synthesised by sintering porous zirconia, magnesia and zirconia/magnesia ceramic samples, designated as ZrO_2_(100), ZrO_2_(75)MgO(25), ZrO_2_(50)MgO(50), ZrO_2_(25)MgO(75), MgO(100) according to their composition, and characterised them by means of microscopy, crystallography, spectroscopy and spectrometry to explore the incorporation of Mg atoms into the zirconia lattice and calculate the porosity of the samples. The final porosity of the cylinder-shaped samples in this study ranged between 30 and 37%. The mechanical analysis exhibited that the Young modulus increased and the microstress decreased with increasing magnesia amount, with values ranging from 175 GPa for zirconia to 301 GPa for magnesia. We observed an increase in adhesion, viability, proliferation and osteogenic activity of MC3T3-E1 pre-osteoblastic cells cultured onto zirconia/magnesia ceramics, with the magnesia-containing ceramics demonstrating higher values of calcium mineralisation. Taken together, the results from the ALP activity, calcium and collagen production clearly show that the zirconia/magnesia ceramics possess robust osteoinductive capacity, therefore holding great potential for bone tissue engineering.

## Data Availability

The data presented in this study are available on request from the corresponding author.

## Supplementary Materials

The following are available online at https://www.mdpi.com/1996-1944/14/4/1049/s1.
