# Supplementary material for: Porous Zirconia/Magnesia Ceramics Support Osteogenic Potential In Vitro
Source: Materials (Basel). 2021 Feb 23;14(4):1049. doi: 10.3390/ma14041049 (PMC7926502; doi:10.3390/ma14041049)
Supplement: Supplementary file 1 [file materials-14-01049-s001.zip › materials-1096736-supplementary.pdf]

materials

MDPI

Article

Porous Zirconia/Magnesia Ceramics Support Osteogenic Potential In Vitro

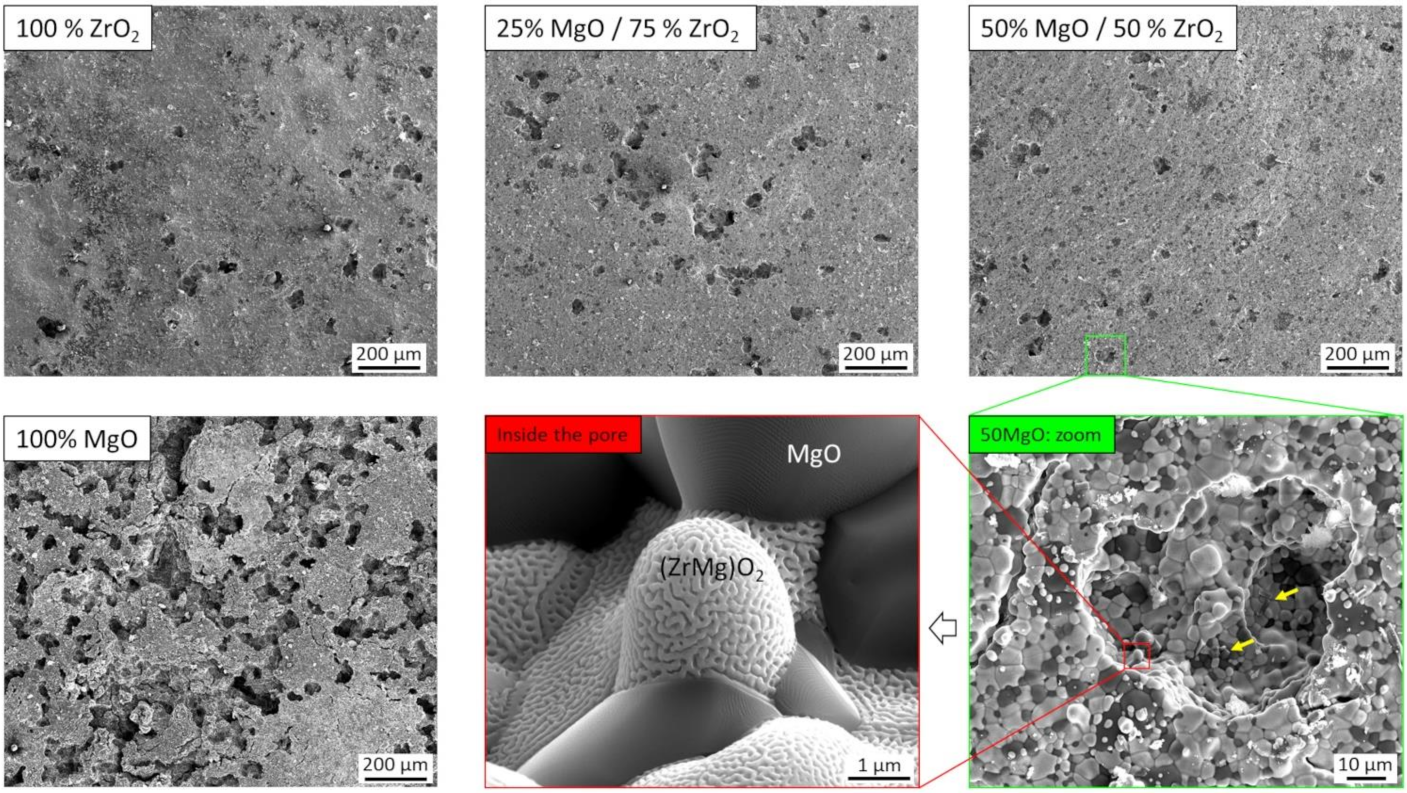

Figure S1. SEM micrographs of ZrO_2/MgO ceramics with a pore size of 50–60 µm. In the samples Figure 2. were detected (see text). In agreement to Scheme 2.

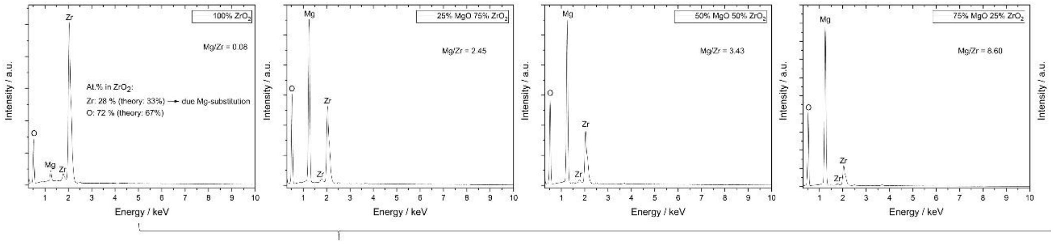

Copyright: © 2021 by the authors. Licensee MDPI, Basel, Switzerland. This article is an open access article distributed under the terms and conditions of the Creative Commons Attribution (CC BY) license (http://creativecommons.org/licenses/by/4.0/).

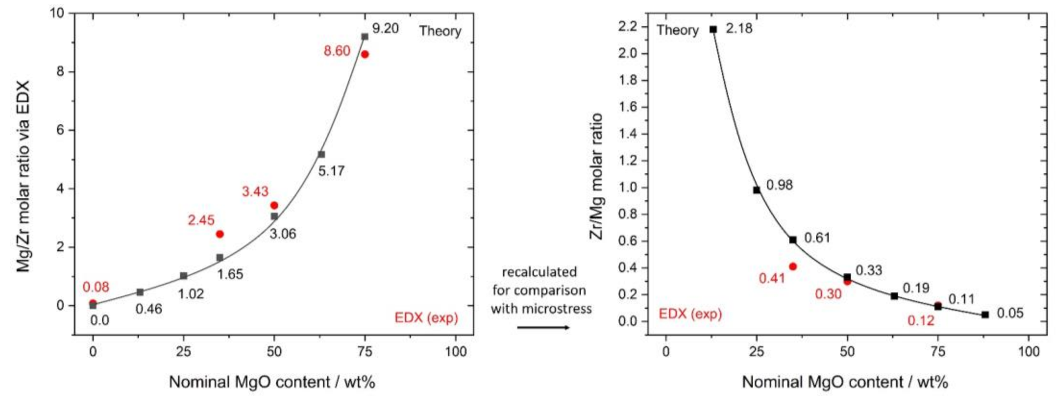

Figure S2. EDX spectra of ZrO_2 / MgO ceramics (**top**) with determined molar Mg/Zr (**bottom**, **left**) and Zr/Mg (**bottom**, **right**) ratios in a good agreement to the nominal compositions and theoretical values.T o verify a slight deviation in the composition (nominal 25 wt.% MgO) from the linear rule determined by XRD (Figure S3, left), AAS measurements of in H_2SO_4 acid dissolved ceramics were performed and the same discrepancy was found (Figure S3, right).

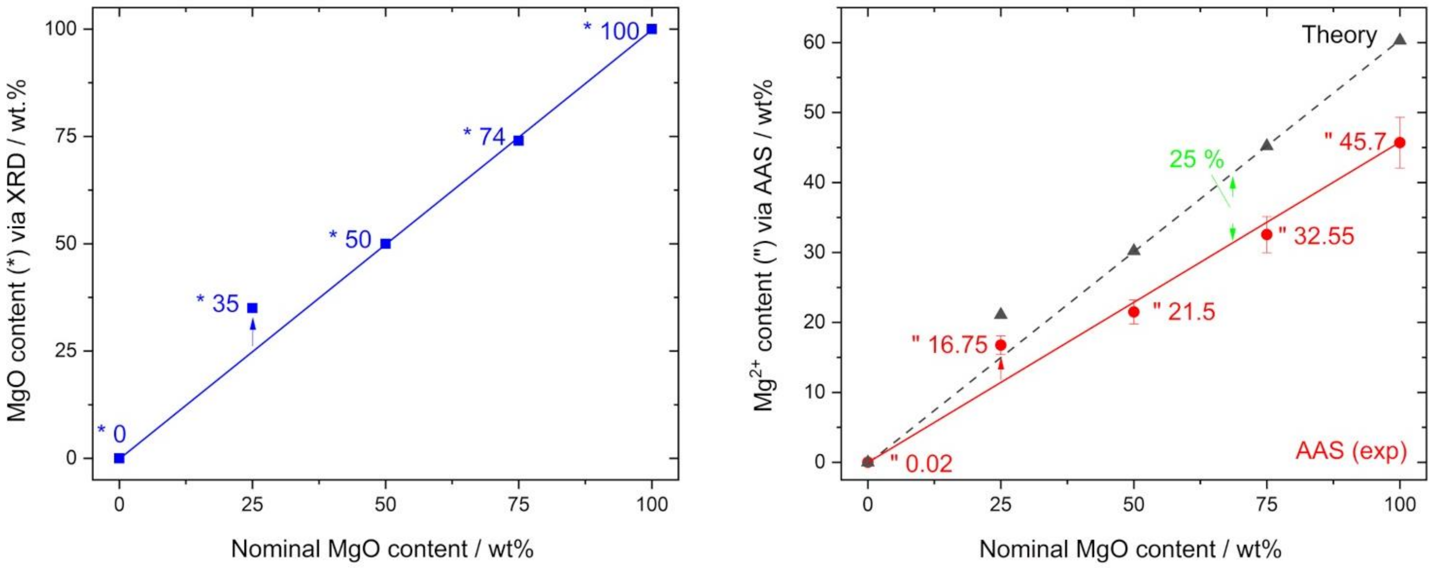

Figure S3. MgO (*) and Mg^{2+} (") content in the ZrO_2 / MgO ceramics was determined by Rietveld refinement in XRD (**left**) and AAS (**right**), respectively. Note: a slight deviation from the linear rule (nominal 25 wt.% MgO) is shown by labeled arrows in both XRD and AAS.
